# Is pornography use a risk for adolescent well-being? An examination of temporal relationships in two independent panel samples

**DOI:** 10.1371/journal.pone.0202048

**Published:** 2018-08-10

**Authors:** Taylor Kohut, Aleksandar Štulhofer

**Affiliations:** 1 Department of Sociology, University of Zagreb, Zagreb, Croatia; 2 Department of Psychology, University of Western Ontario, London, Ontario, Canada; University of Westminster, UNITED KINGDOM

## Abstract

Cross-sectional evidence suggests that pornography use is related to lower mental well-being among adolescents but it remains unclear if changes in well-being are related to the dynamics of pornography use within this population. We examined the relationship between pornography use, subjective well-being, symptoms of depressions and anxiety, and self-esteem in two independent panel samples (*N* = 455; *N* = 858) of Croatian adolescents using cross-lagged path analysis and lagged linear mixed models. After controlling for impulsiveness and family environment—factors that are unlikely to be influenced by pornography use—earlier levels of pornography use were not significantly associated with subsequent decreases in subjective well-being across gender and panel. However, pornography use was associated with increases in both self-esteem and symptoms of depression and anxiety, albeit only among adolescent women in one of the two panels. In addition, low subjective well-being was associated with a subsequent increase in pornography use, but only in female adolescents in one panel. This study’s results are not consistent with concerns about pornography use negatively contributing to male adolescents’ psychological well-being, but suggest potential antagonistic links between pornography use and specific facets of mental well-being in adolescent women. Such links should be considered tentative until verified with further research.

## Introduction

Concern about children’s use of pornography has a long history that extends back to the Victorian era [1]. However, the rise of Internet pornography has given new urgency to this issue, presumably because of the increased anonymity, affordability, and accessibility [2,3] that it is said to provide [4]. In our current age, popular media discussions of the dangers of Internet pornography for children and adolescents have begun to revolve around public health conceptualizations [5]. In some cases, receptive governments have responded accordingly, with Utah being the first of several American states to declare pornography a “public health crisis” [6], Canada commissioning a Parliamentary study on the public health effects of violent and degrading pornography [7], and the UK implementing increasingly restrictive regulation of Internet pornography, despite admissions by regulatory bodies that no clear harms to children have been demonstrated [8].

Though sampling and methodological issues preclude precise prevalence estimates [9–11], it is believed that 7–59% of adolescents are accessing pornography intentionally [12]. Given the non-trivial rates of pornography use among teens, there are several reasons for concern about the impact of Internet pornography on adolescent development. Specifically, some believe that pornography may impact adolescents’ sexual risk taking, sexual functioning, body image, sexual objectification and sexual aggression [12–15]. From this perspective, Internet pornography may threaten many facets of adolescent development and well-being, particularly because “children and adolescents are widely considered the most vulnerable audiences to sexually explicit material”[15].

Of particular interest are the implications of potential harms of pornography on adolescent well-being. At an individual level, well-being refers to a state of mental and physical wellness and involves both objective and subjective components [16]. In the social sciences, mental well-being has been further differentiated into the interrelated concepts of subjective well-being (the hedonic tradition) and psychological well-being (the eudaimonic tradition)[17,18]. Subjective well-being is generally conceptualized as the experience of positive, rather than negative affect, combined with a sense of life satisfaction [19]. In contrast, psychological well-being, which was inspired by more humanistic philosophies, conceptualizes well-being along six dimensions, including self-acceptance, positive relations with others, autonomy, environmental mastery, purpose in life, and personal growth [20]. The literature concerning pornography use and mental well-being stems from the study of late adolescents and early adulthood, and can be largely organized around the domains of self-evaluations, interpersonal functioning, and the experience of dysregulated affect.

Although research findings are mixed, there are several reasons to believe that pornography use may impact the mental well-being of adolescents. For example, pornography use may contribute to personal insecurities about adolescents’ bodies, their appearance, or their sexual performance [13,21,22] and it may undermine attachment functioning, leading to relationship dysfunction, and social isolation [15,23,24]. Furthermore, cross-sectional surveys have found that pornography use is related to reports of more negative affect, poor mental health and lower quality of life among adults [24,25] as well as lower life-satisfaction and self-esteem, and more symptoms of depression among adolescents [12,26–28].

On the basis of this evidence, the case for pornography having a negative impact on adolescent mental well-being may seem strong, however, several studies fail to support this conclusion—at least partially. For example, some research has indicated that pornography use is either unrelated, or positively related to body and genital satisfaction, and to sexual esteem among adult samples [22,24,29]. Further, other studies have failed to observe significant relationships between social connectedness, attachment to parents and peers, and pornography use among adolescents [11,12], or have found that adult pornography users actually have more close relationships than non-users [30]. Finally, at least one study has failed to find a significant association between pornography use and self-esteem among adolescents [12], while another reported a positive relationship between the two constructs in young adult males [29].

Even if one were to assume that the bulk of accumulated evidence favors the hypothesis that pornography use is associated with lower mental well-being among adolescents, it still remains unclear if pornography use can *cause* impairments in well-being. The primary issue is the failure to control for potential confounds, or variables that may reasonably be expected to create spurious correlations between pornography use and psychological health. For example, it is unlikely that pornography use among adolescents causally contributes to impulsivity and poor family functioning—although these characteristics have been found to be associated with pornography use [12,31] and are also likely connected to poor mental well-being. A failure to control for such variables may contribute to the conflicting findings discussed above.

Secondly, despite causal reasoning that underlies many theories that are employed when studying the presumed effects of pornography use (e.g., Social Cognitive theory, Sexual Scripting theories, Social Comparison theory, etc), the vast majority of research in this domain has employed cross-sectional designs. Although growing in number, there are still relatively few longitudinal studies of pornography use among adolescents which are helpful for uncovering evidence of antecedent order, and—for obvious ethical reasons—no experimental studies (that we are aware of).

Finally, assuming that pornography use is responsible for impaired well-being ignores the possibility that pornography, as an entertainment medium, may be used intentionally to improve mood or adjust poor psychological health states. When pornography users are asked why they use pornography, aside from its obvious sexual gratification function, many people report using pornography to induce positive affect (e.g. use for entertainment) or to alleviate negative affective states such as boredom, stress, or depression [32–35], suggesting that negative mental states can precede, rather than follow, pornography use. Substantiating this possibility further, the only longitudinal analysis of the connection between pornography use and subjective well-being among adolescents reported that low life satisfaction predicted subsequent increases in pornography use over time [11]. Such evidence challenges unidirectional causal thinking in favor of transactional theories, such as the Differential Susceptibility to Media Effects model [36], which articulate the causal interplay between pornography use and its presumed harms over time.

### The current study

To address shortcomings in our understanding of the relationship between pornography use and mental well-being among adolescents, we used two independent panel samples of Croatian adolescents to explore the following research question (informed by the current weight of evidence): Are the dynamics of frequency of pornography use associated with the dynamics of subjective well-being among adolescents? Given the diverse findings and the conceptual and methodological limitations in this area, the association between pornography use and a global measure of subjective well-being was explored using cross-lagged structural equation modeling approach with two time-points 12 months apart. This relatively lengthy period of time under observation provides insights into possible longer-term effects of exposure to sexually explicit imagery.

To supplement these analyses, additional associations between pornography use and two facets of subjective well-being—dysregulated affect (operationalized as symptoms of depression and anxiety) and self-esteem—were examined with a linear mixed model approach to lagged analysis. Although self-esteem is more clearly affiliated with the conceptual definition of psychological rather than subjective well-being [19,20], empirical evidence indicates that self-esteem is moderately to strongly associated with global measures of subjective well-being [37,38], indicating that self-evaluations may be a point of overlap between the two concepts. Examining these facets offered the opportunity to consider finer-grain associations between pornography use and mental well-being, and helped connect our analysis of subjective well-being to the existing body of evidence concerning pornography use, mental health, and self-evaluations.

## Method

### Participants and procedures

The data for this study were collected in two panel samples of Croatian adolescents from Zagreb and Rijeka that were recruited as a part of the PROBIOPS (Prospective Biopsychosocial Study of the Effects of Sexually Explicit Material on Young People’s Sexual Socialization and Health) project. The samples included high-school sophomores (*M*_Zagreb_ = 16.1 years, SD = 0.46, range = 15–19 and *M*_*Rijeka*_ = 15.9 years, SD = 0.52, range = 15–18) who were then re-surveyed at 6-month intervals (baseline surveys were conducted in April of 2015 in Zagreb and December the same year in Rijeka). In Zagreb, students were recruited from 59 of 90 schools in the capitol city and the surrounding county. In Rijeka, the panel included students from 14 larger secondary schools, which accounted for 63% of the city’s 2^nd^ year high-school student population.

Unlike the Zagreb panel, which was carried out using Internet surveying (after 2,655 adolescents registered online to receive a unique code), the Rijeka sample was classroom based, with screens placed between students to maximize confidentiality. The attrition rate was substantially higher in Zagreb despite the use of incentives (a lottery with $7–14 vouchers was organized at each data collection wave). The number of participants in the Zagreb panel dropped sharply from baseline (*n* = 2,241) to the second wave (*n* = 644), after which it stabilized. In the Rijeka panel, in which participants received no compensation for participation, attrition was mostly due to school absenteeism and mistakes in re-creating a 5-digit alphanumeric identification code at each wave. The panel size varied from 1,291 at wave 1 (W1) to 1,177 at W4. At W5, it dropped to 931 participants because students enrolled in 3-year vocational schools finished their secondary education between W4 and W5.

The analyses involving subjective well-being relied on data from *n* = 123 male and *n* = 332 female adolescents (*N* = 455) from the Zagreb panel, who participated at W3, W4 and W5, and *n* = 326 male and *n* = 532 female students (*N* = 858) from the Rijeka panel, who participated at W1, W2 and W4. The selection was based on the fact that some of the key constructs were measured only at these waves. To address possible attrition bias, a multivariate logistic regression analysis was carried out by panel, with the dependent variable denoting adolescents who were included in this study (coded 1) and those who were not (coded 0). In addition to baseline frequency of pornography use, several sociodemographic indicators (gender, father’s and mother’s education, academic achievement and religiosity) were also included. In Zagreb, adolescent women (*AOR* = 2.65, *p* < .001) and students with higher academic achievement (*AOR* = 1.79, *p* < .001) had higher odds of being included in this study. In Rijeka, only academic achievement differed significantly between the two groups of students (*AOR* = 1.46, *p* < .001). According to recent guidelines, these differences should be interpreted as small in size [39].

Analyses involving depression and anxiety and self-esteem, indicators of subjective well-being, were based on different subsets of data from the two panels. In this case, data was provided by *n* = 200 male and *n* = 443 female adolescents (*N* = 643) from the Zagreb panel, who participated in at least two of the three relevant data collection waves (W2-W4), and *n* = 468 male and *n* = 711 female students (*N* = 1,179) from the Rijeka panel, who participated in at least three of the five waves. To address possible attrition bias, a multivariate logistic regression analysis was carried out by panel, with the dependent variable denoting adolescents who were included in this study (coded 1) and those who were not (coded 0). In addition to baseline frequency of pornography use, self-esteem and depression/anxiety symptoms (this variable was not measured at baseline in the Zargeb panel), several sociodemographic indicators (gender, father’s and mother’s education, academic achievement and religiosity) were also included. In Zagreb, adolescent women (*AOR* = 2.04, *p* < .001) and students who reported higher academic achievement (*AOR* = 1.84, *p* < .001) were characterized by higher odds of being included in this study compared to other participants in the panel. In the Rijeka panel, female students (*AOR* = 1.82, *p* < .05), participants with higher academic achievement (*AOR* = 2.08, *p* < .001), and those with more educated mothers (*AOR* = 1.41, *p* < .05) had significantly higher odds of being included in this study. Again, the observed differences were small [39].

According to the national guidelines for ethical research in minors, which stipulate that informed consent can be sought of adolescents aged ≥ 14 years[40], parents were sent a letter with information about the study prior to its launch and consent was sought directly from adolescents. In the online panel (Zagreb), participants confirmed their consent (after reading detailed information about the study and participation) by clicking on a button. Only participants who provided informed consent were able to access the questionnaire. The procedure was repeated in each wave. In the classroom-based panel (Rijeka), information required for informed consent was provided orally (by a study assistant) and in print, on the first page of the questionnaire booklet. Students were instructed to read the information carefully and then proceed to the questionnaire only if they understood the information and agreed to be a participant in the study.

In addition to consent-related information, all questionnaires contained the contact information of a national organization that offers support and counseling to children and young people. In Rijeka, the informed consent procedure was also communicated by a research assistant who supervised classroom surveying. The ethical research board of the University of Zagreb approved all study procedures, including our consent procedures and the lack of parent/guardian consent for participants older than 14 years of age. Permission was obtained from all participating schools before the participant recruitment began. The rationale, hypotheses, and data-analytic plan were developed before analyzing data from the Zagreb panel. Confirmatory predictions were then pre-registered on the Open Science Framework prior to data analysis taking place with the Rijeka panel data (see https://osf.io/ajqg4/?view_only=a1ffcf1871234f4ea8a9c2def2a487ee).

### Measures

#### Pornography use

The frequency of pornography use was assessed at each wave with the item, “How often have you used pornography during the last 6 months?” In the questionnaire, pornography was defined for participants as *any material which openly depicts sexual activity; material which shows naked bodies but not sexual intercourse or other sexual activity does not belong to pornography as here defined*. Response options included: 1 = not once, 2 = several times a month, 3 = once a month, 4 = 2–3 times a month, 5 = once a week, 6 = several times a week, 7 = every day or almost every day, and 8 = several times a day. Stability coefficients for the indicator were in the *r* = .68-.83 range. At baseline, 49.70% of the Zagreb sample and 55.69% of the Rijeka sample reported pornography use.

#### Subjective well-being

Defined as a highly personal assessment of quality of life [41], subjective well-being was measured at W3 and W5 in the Zagreb panel and W2 and W4 in Rijeka. In the corresponding analyses, these time points are referred to as T1 and T2. We used an adapted 4-item version of the Personal Well-being Inventory—School Children (PWI-SC) [42] to indicate participants’ well-being. The four items asked about satisfaction with various facets of life, including health, relationships and personal achievement. Responses were collected with 10-point scales that ranged from 1 = completely unsatisfied to 10 = completely satisfied. Reliability of this measure was satisfactory in both the Zagreb (Cronbach’s α = .80 and .81) and Rijeka samples (Cronbach’s α = .81 and .84), as was its stability across the period of 12 months (*r* = .74 and .66, respectively).

#### Depression and anxiety

This construct was assessed using the Patient Health Questionnaire for Depression and Anxiety (PHQ-4)[43] at W2-W4 in the Zagreb panel and W1-W5 in the Rijeka panel, This brief 4-item screening scale that asked about the symptoms of depression (2 items) and anxiety (2 items) experienced in the two weeks preceding the survey. The frequency of symptoms was measured on a 4-point scale ranging from 1 = not at all to 4 = nearly every day. The measure had satisfactory reliability (Cronbach’s α ranged were in the .83 to .86 range) and reasonable stability (*r* = .51 to .64).

#### Self-esteem

General self-esteem was assessed by a 4-item scale (e.g., “In general, I like myself the way I am” and “When I do something, I do it well”) used in a longitudinal study of Canadian teenagers [44]. This measure was implemented in W2-W4 in the Zagreb panel and W1-W5 in the Rijeka panel. A Likert-like scale, ranging from 1 = it doesn’t relate to me at all to 5 = it completely relates to me, was employed to anchor responses. The indicator had satisfactory reliability (Cronbach’s α = .81 to.84) and stability (r = .59 to .74) in this study.

#### Impulsiveness

The construct was measured with an adapted 6-item version of the 7-item unidimensional Barratt Impulsiveness Scale-Brief (BIS-Brief)[45] at W4 in Zagreb and W2 in Rijeka. The construct was treated as a time invariant characteristic (personal trait). Example items included: “I don’t pay attention, I act on the spur of the moment,” and “I do things without thinking.” Responses were collected on 4-point scales ranging from 1 = never or rarely to 4 = almost always or always. Following exploratory factor analysis which resulted with a 2-dimensional structure in each panel (the second dimension included the only two reverse worded items; e.g., *I concentrate easily*), pairing of the items in three parcels for structural equation modeling was guided by their dimensionality. The measure’s reliability was satisfactory in both panels (Cronbach’s α was .73 in the Zagreb and .75 in the Rijeka panel).

#### Adverse family environment

This construct, focused on hostile and/or aggressive family atmosphere, was assessed at W4 in Zagreb and W1 in Rijeka by three items that asked about the frequency of intense domestic quarrels, aggressive behaviors and family members being systematically ignored in the past 12 months. A 5-point scale ranging from 1 = never to 5 = often was used to anchor answers. The three items were summed into a composite measure with an acceptable internal consistency (Cronbach’s *α*_Zagreb_ = .71 and *α*_Rijeka_ = .72). Higher scores denoted more adverse family environment.

All measures used in this study were presented in Croatian and were characterized by identical wording and formatting across the two panels. The original Croatian wording and English translations of all measures can be found in S1 Appendix.

### Analytical strategy

With the two independent panels employing the same measures, albeit, often measured in different waves, the overarching data analytic plan was to compare the results of conceptually similar analyses across the two samples in an exploratory-confirmatory fashion. Thus, the interpretation of our findings rested primarily on consistent patterns of null and significant associations across the Zagreb and Rijeka panels, though inconsistent findings were also noted. Because of differences in the availability of subjective well-being, depression and anxiety, and self-esteem across measurement occasions, two separate cross-lagged models were initially pre-registered: one involved subjective well-being (described below) while the other incorporated both depression and anxiety and self-esteem. Following preliminary data analysis, the cross-lagged model involving pornography use, depression and anxiety, and self-esteem was supplanted by a lagged linear mixed model approach described below.

#### Cross-lagged models with well-being

The association between pornography use and subjective well-being was explored with cross-lagged path analytic models, with pornography use at first measurement (T1) predicting well-being in the next measurement (T2) and vice versa. It should be noted that the assessment likely covered somewhat different developmental phases in the two panels. In Rijeka, participants were about 16.5 years at T1 and 17.5 at T2, while in the Zagreb panel the respective ages were approximately 17.5 (T1) and 18.5 (T2).

The analysis was carried out in several steps [46] and began with data from the Zagreb panel, which were used to pre-register expectations for the Rijeka panel. First, an unconditional cross-lagged model (Model 1) was explored. Error variances of the two constructs were allowed to co-vary between T1 and T2, as well as error terms of each well-being item. In the next step, impulsiveness and adverse family environment were added to the model as correlates of pornography use and well-being at both measurements to control for possible confounding. This final model (Model 2) was then explored for gender invariance. Using a multi-group comparative approach, tests were carried out in progressively more restrictive steps, from configural to strong factorial invariance [46]. Taking into account a relatively large sample size of the Rijeka panel, the standard χ^2^ difference test (which was used in the Zagreb panel) was replaced with ΔCFI test. Values ≤ .002 indicated a non-significant difference between less and more constrained models [47]. Due to measurement discrepancies in impulsiveness, only configural invariance was confirmed in the Zagreb panel and weak factorial invariance in the Rijeka panel, which suggested that direct comparisons between male and female adolescents should be avoided in both panels. Guided by standard guidelines for longitudinal assessment [46], model fit was evaluated at each step based on *χ*^2^/df ratio, comparative fit index (CFI) and the root mean square error of approximation (RMSEA). Adequate fit to the data was indicated by *χ*^2^/df ratio ≤ 2, CFI values ≥ .95 and RMSEA values ≤ .05 (with the upper 90% confidence interval end ≤ .08).

The robustness of findings was checked by bootstrapping Model 2 in both panels with 2,000 resamples to address multivariate non-normality. With the exception of adolescent men’s model in the Zagreb panel (which failed to converge, most likely because of the small sample size), the pattern of significant findings remained unchanged.

Given that less than 2% of information was missing in the Zagreb panel and < 5% in the Rijeka panel, full information maximum likelihood estimation was used to handle missing values [48]. Due to the fact that nestedness in schools (Zagreb) or classes (Rijeka) explained a small proportion of variance in participants’ pornography use (< 5%) and subjective well-being (5% in the Zagreb and 9.3% in the Rijeka panel), the effects of intra-class correlation were disregarded in path analysis.

#### Lagged linear mixed models

Initially, associations between pornography use, depression and anxiety, and self-esteem were tested using separate cross-lagged models by panel and the data and syntax for these analyses are available online (https://osf.io/cbz5y/?view_only=7de14725f32748dfa0cd99b56e9240b1). Unexpected difficulties arose in the interpretation of the results. Problems included the failure to replicate significant paths and covariances across the two models, the failure to replicate significant paths and covariances across measurement occasions within the same models, and inconsistent directions (i.e., a mixture of positive and negative coefficients) in significant paths between pornography use and self-esteem. To clarify the findings, a post-hoc decision was made to re-examine the associations between these variables using a linear mixed modeling approach to lagged analysis. This is a generalized approach to lagged regression analysis where a dependent variable recorded at waves *t* + 1 is regressed on an independent variable at waves *t* while controlling for the level of the dependent variable at waves *t*. Importantly, dependencies in the residuals are also modeled with heterogeneous auto-regressive structure (ARH1), while any further nesting can be accounted for with random effects. In this analysis, a significant effect for the independent variable indicates average associations between the independent variable and changes in the dependent variable across all waves in a given panel. Essentially, this approach provides information that is similar in interpretation to path coefficients in a cross-lagged model but aggregated across measurement occasions.

For each panel, this approach was applied separately to the prediction of depression and anxiety, self-esteem, and pornography use. For example, pornography use, depression and anxiety, and self-esteem at waves *t* were used to predict depression and anxiety at waves *t* + 1 while modeling residuals with an ARH1 structure and including a random intercept for schools in the Zagreb panel and classes in the Rijeka panel. In several cases, models that were tested with Zagreb panel data would not converge due to low variability in the intercept for schools. In these cases, the random effect was fixed to zero. In the final step, impulsiveness and adverse family environment were added to each model to examine the robustness of effects that emerged for pornography use while controlling for the two possible confounders.

## Results

Consistent associations between pornography use and subjective well-being were limited to adolescent women (see Table 1), and similar links between pornography use and depression and anxiety were only found among women in the Rijeka panel (see Tables 2 and 3). Correlations between pornography use on the one hand and depression/anxiety and self-esteem on the other hand were largely non-significant among adolescent men in both panels, as well as for adolescent women in the Zagreb panel. Among females in the Rijeka panel, the associations were inconsistent, with relationships between pornography use and depression/anxiety symptoms, but not self-esteem, mostly significant. Links between the focal variables and hypothesized confounders (impulsiveness and adverse family environment) were mostly significant and in the expected directions.

**Table 1:** Associations between the focal variables used in the subjective well-being analysis.

	1	2	3	4	5	6	*M* (SD)
1) Pornography use at T1		.70** .66**	-.09 -.06	-.09 -.12*	.02 .15**	.14 .00	Male participants = 5.42 (1.90) Female participants = 2.38 (1.83) *Rijeka* Male participants = 4.98 (2.15) Female participants = 1.76 (1.38)
2) Pornography use at T2	.70** .67**		-.08 -.16**	-.18* -.16**	-.07 .12*	.12 -.02	*Zagreb* Male participants = 5.72 (1.61) Female participants = 2.59 (1.87) *Rijeka* Male participants = 4.89 (2.13) Female participants = 1.92 (1.56)
3) Well-being at T1	.04 -.18**	.03 -.15**		.76** .75**	-.22* -.30**	-.08 -.18**	*Zagreb* Male participants = 31.10 (6.79) Female participants = 29.78 (6.18) *Rijeka* Male participants = 33.58 (5.44) Female participants = 31.53 (5.96)
4) Well-being at T2	.01 -.15**	-.07 -.15**	.63** .66**		-.20* -.29**	-.22* -.22**	*Zagreb* Male participants = 31.58 (5.66) Female participants = 30.11 (6.14) *Rijeka* Male participants = 33.07 (5.85) Female participants = 31.45 (6.07)
5) Adverse family environment	.10 .16**	.12* .13**	-.29** -.27**	-.22** -.27**		.24** .22**	*Zagreb* Male participants = 4.35 (1.41) Female participants = 5.00 (2.04) *Rijeka* Male participants = 4.27 (1.56) Female participants = 4.75 (1.86)
6) Impulsiveness	.21** .11**	.22** .09*	-.33** -.33**	-.22** -.30**	.19** .20**		*Zagreb* Male participants = 15.80 (3.74) Female participants = 16.59 (3.93) *Rijeka* Male participants = 16.35 (4.16) Female participants = 17.12 (4.27)

Zero-order coefficients for the Zagreb panel are presented above the main diagonal and those for the Rijeka panel are shown below it; coefficients for male participants are in the top row and coefficients for their female peers in the bottom row

* *p* < .05

** *p* < .01

*** *p* < .001

**Table 2:** Associations between the focal variables used in analyses of depression and anxiety and self-esteem for the Zagreb panel (n = 200 males; n = 443 females).

												Male		Female	
	1	2	3	4	5	6	7	8	9	10	11	*M*	*SD*	*M*	*SD*
Pornography Use W1 (1)	—	.72***	.55***	.08	.01	-.01	.02	.05	-.02	.2**	.14	5.32	2.06	2.27	1.84
Pornography Use W2 (2)	.70***	—	.73***	.08	.06	.07	-.04	-.09	-.05	.16*	.08	5.31	2.04	2.29	1.77
Pornography Use W3 (3)	.65***	.73***	—	.02	.07	.05	-.03	-.06	-.05	.10	.08	5.26	1.98	2.36	1.81
Depression and Anxiety W1 (4)	.05	.08	.06	—	.65***	.54***	-.40***	-.36***	-.36***	.18*	.33***	8.23	2.74	10.25	3.27
Depression and Anxiety W2 (5)	.06	.04	.08	.56***	—	.64***	-.17*	-.29***	-.25**	.13	.33***	8.25	3.00	9.93	3.11
Depression and Anxiety W3 (6)	.05	.09	.13**	.53***	.60***	—	-.22*	-.23**	-.31***	.23**	.32***	7.87	2.77	9.68	3.30
Self-Esteem W1 (7)	.00	.00	.01	-.37***	-.37***	-.31***	—	.60***	.66***	-.20*	-.17*	15.99	2.66	15.19	2.90
Self-Esteem W2 (8)	.00	-.04	-.07	-.30***	-.48***	-.36***	.75***	—	.65***	-.21*	.21**	16.05	2.79	14.83	3.19
Self-Esteem W3 (9)	.07	-.01	-.02	-.32***	-.39***	-.40***	.68***	.68***	—	-.23**	-.09	15.87	2.89	14.84	3.12
Impulsiveness (10)	-.00	-.01	.02	.15**	.16**	.15**	-.18***	-.20***	-.23***	—	.18*	15.82	3.78	16.72	3.95
Adverse Family Environment (11)	.13*	.16***	.14**	.34***	.27***	.36***	-.22***	-.26***	-.22***	.20***	—	4.36	1.31	5.13	1.79

Correlations for males lie above the diagonal while correlations for females lie below the diagonal

* signifies significant correlations p < .05

** signifies significant correlations, p < .01

*** signifies significant correlations, p < .001

**Table 3:** Associations between the focal variables used in analyses of depression and anxiety and self-esteem for the Rijeka panel (*n* = 468 males; *n* = 711 females).

																		Male		Female	
	1	2	3	4	5	6	7	8	9	10	11	12	13	14	15	16	17	*M*	*SD*	*M*	*SD*
Pornography Use W1 (1)	—	.70***	.63***	.57***	.52***	.02	.04	.06	.07	.05	-.02	.01	.01	-.00	.08	.19***	.13*	4.78	2.17	1.73	1.38
Pornography Use W2 (2)	.68***	—	.77***	.70***	.60***	.01	.08	.10	.08	.02	.02	-.05	.01	-.02	-.02	.21***	.04	5.01	2.14	1.79	1.42
Pornography Use W3 (3)	.60***	.72***	—	.77***	.68***	.01	.05	.09	.11*	.02	-.05	-.08	-.02	-.11*	-.07	.19***	.05	4.72	2.17	1.76	1.38
Pornography Use W4 (4)	.56***	.67***	.68***	—	.73***	.00	.10*	.11*	.09	.09	-.03	-.04	-.04	-.05	-.07	.23***	.09	4.88	2.13	1.90	1.54
Pornography Use W5 (5)	.57***	.65***	.69***	.77***	—	.03	.03	.03	.09	.09	-.03	-.07	-.04	-.03	-.01	.22***	.14*	5.12	2.04	1.89	1.48
Depression and Anxiety W1 (6)	.14***	.14***	.15***	.12**	.11*	—	.55***	.54***	.47***	.47***	-.21***	-.18***	-.22***	-.19***	-.15*	.22***	.27**	7.72	2.81	9.52	3.33
Depression and Anxiety W2 (7)	.17***	.15***	.15***	.10*	.10*	.64***	—	.57***	.52***	.45***	-.23***	-.27***	-.18***	-.24***	-.23***	.24***	.17***	7.35	2.62	8.77	3.07
Depression and Anxiety W3 (8)	.22***	.15***	.16***	.17***	.15**	.52***	.58***	—	.58***	.50***	-.24***	-.25***	-.25***	-.29***	-.28***	.23***	.40***	7.22	2.57	8.31	2.89
Depression and Anxiety W4 (9)	.12**	.10*	.14**	.11**	.13**	.47***	.54***	.60***	—	.52***	-.15**	-.17**	-.27***	-.28***	-.26***	.22***	.24***	7.31	2.54	8.71	3.05
Depression and Anxiety W5 (10)	.20***	.17***	.25***	.17***	.13**	.47***	.54***	.61***	.56***	—	-.10	-.13*	-.19**	-.19**	-.17**	.27***	.27***	6.95	2.58	8.32	2.95
Self-Esteem W1 (11)	-.16***	-.12**	-.17***	-.06	-.14**	-.33***	-.32***	-.28***	-.25***	-.29***	—	.72***	.63***	.66***	.51***	-.32***	-.26***	16.50	2.49	15.22	2.88
Self-Esteem W2 (12)	-.13**	-.10**	-.10*	-.06	-.07	-.37***	-.34***	-.32***	-.27***	-.28***	.72***	—	.66***	.65***	.54***	-.35***	-.17***	16.73	2.54	15.47	2.95
Self-Esteem W3 (13)	-.06	-.04	-.08	-.07	-.04	-.22***	-.26***	-.34***	-.25***	-.24***	.65***	.71***	—	.68***	.59***	-.31***	-.24***	16.18	2.51	15.46	2.68
Self-Esteem W4 (14)	-.11**	-.07	.09*	-.04	-.08	-.28***	-.30***	-.33***	-.35***	-.28***	.67***	.69***	.71***	—	.69***	-.28***	-.11*	16.65	2.36	15.73	2.71
Self-Esteem W5 (15)	.08	-.09	-.12*	-.02	-.09*	-.25***	-.28***	-.34***	-.30***	-.39***	.64***	.66***	.70***	.76***	—	-.25**	-.11	16.33	2.49	15.83	2.77
Impulsiveness (16)	.20***	.17***	.18***	.17**	.15***	.24***	.30***	.29***	.23***	.30***	-.37***	-.37***	-.36***	-.33***	-.40***	—	.22***	17.11	3.64	18.14	3.71
Adverse Family Environment (17)	.20***	.23***	.27**	.19***	.21***	.29***	.30***	.27***	.24***	.28***	-.16***	-.14***	-.15***	-.16***	-.15**	.29***	—	4.14	1.37	4.61	1.58

Correlations for males lie above the diagonal while correlations for females lie below the diagonal

* signifies significant correlations p < .05

** signifies significant correlations, p < .01

*** signifies significant correlations, p < .001

### Pornography use and well-being

A cross-lagged path analytic model was used to explore the direction of associations between adolescent pornography use and subjective well-being, while controlling for their previous levels. Among male adolescents in the smaller panel (Zagreb), neither of the two direct paths were significant, but we observed a negative covariance (*cov* = -0.26, S.E. = 0.10, *p* = .009) between the two constructs at T2. Among their female peers, two paths were significant: the one leading from pornography use at T1 to well-being measured at T2 (*b* = -0.06, S.E. = 0.03, *p* = .047) and the path from well-being at T1 to pornography use at T2 (*b* = -0.15, S.E. = 0.05, *p* = .005). While a higher baseline frequency of adolescent women’s pornography use was associated with a lower reported well-being 12 months later, lower initial levels of well-being were related to higher subsequent pornography use.

The pattern of findings in the larger panel (Rijeka) was identical in the male but not female sample. Among adolescent men, we again found a significant covariance between pornography use and well-being at T2 (*cov* = -0.25, S.E. = 0.11, *p* = .018). With respect to female adolescents, no significant cross-lagged paths were observed. In further contrast to the Zagreb findings, a negative and significant covariance between the two constructs was found at T1 (*cov* = -0.33, S.E. = 0.09, *p* < .001).

**Fig 1:**
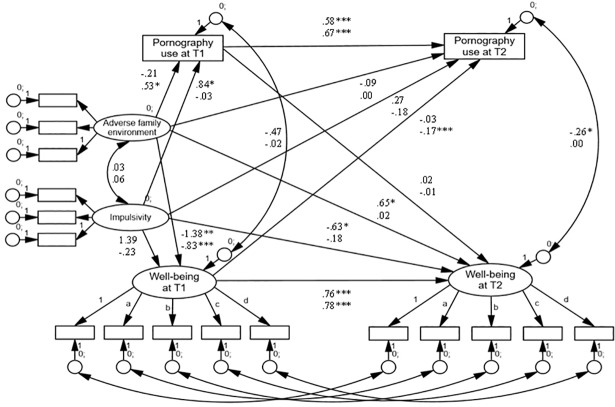
Final cross-lagged path model (Model 2) of the association between adolescent pornography use and well-being in the Zagreb panel. *Notes*. Model fit: *χ*^2^_(254)_ = 436.30, CFI = .944, RMSEA = .040; unstandardized paths and covariances in the male sample are presented in the top row and those in the female sample in the bottom row. * *p* < .05, ** *p* < .01, *** *p* < .001.

**Fig 2:**
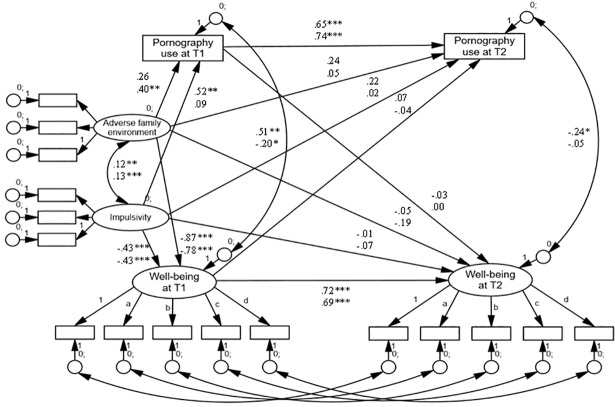
Final cross-lagged path model (Model 2) of the association between adolescent pornography use and well-being in the Rijeka panel. *Notes*. Model fit: *χ*^2^_(262)_ = 583.26, CFI = .945, RMSEA = .038; unstandardized paths and covariances in the male sample are presented in the top row and those in the female sample in the bottom row. * *p* < .05, ** *p* < .01, *** *p* < .001.

Next, to address possible confounding, the basic cross-lagged model was extended by including impulsiveness and adverse family environment as potential confounders. The extended model confirmed the findings in the male sample from Zagreb. Among their female peers, only the path between baseline well-being and subsequent pornography use remained significant (*b* = -0.17, S.E. = 0.06, *p* = .005) (Fig 1). In Rijeka, controlling for the two characteristics resulted in a new significant covariance between well-being and pornography use at T1 among male participants (*cov* = 0.51, S.E. = 0.16, *p* = .002) (Fig 2). In this case, higher pornography use was associated with higher reported well-being, unlike the relationship observed at T2. The change indicated a suppression effect. In the female sample, the covariance at T1 was notably attenuated but remained significant (*cov* = -0.19, S.E. = 0.09, *p* = .028).

As shown in Table 4, all four cross-lagged path models (with and without confounders) had satisfactory fit to data. As a robustness check, but also to aid with multivariate non-normality, the final model was bootstrapped. With the exception of male participants in the Zagreb panel (the analysis failed to converge in this sample), the procedure did not affect the pattern of significant findings.

**Table 4:** Path-analytic models’ fit.

	Zagreb				Rijeka			
	Multi-group model A		Multi-group model B		Multi-group model A		Multi-group model B	
	Adolescent men (*n* = 123)	Adolescent women (*n* = 332)	Adolescent men (*n* = 123)	Adolescent women (*n* = 332)	Adolescent men (*n* = 326)	Adolescent women (*n* = 532)	Adolescent men (*n* = 326)	Adolescent women (*n* = 532)
*χ*^2^ (df)	235.63 (109)		436.30 (254)		345.67 (109)		565.55 (254)	
CFI	.956		.944		.951		.947	
RMSEA (90% CI)	.051 (.042-.060)		.040 (.033-.046)		.050 (.044-.056)		.038 (.034-.042)	

### Pornography use, depression and anxiety, and self-esteem

A linear mixed modeling approach to lagged analysis was employed to determine if pornography use at a given wave was related to subsequent changes in dysregulated mood. When controlling for earlier depression and anxiety and self-esteem, pornography use was not significantly associated with later depression and anxiety among male participants in the Zagreb panel, *b* = -0.02, *p* = .693. In the Rijeka panel, this relationship was significant, *b* = 0.05, *p* = .037. After impulsiveness and adverse family environment were added to the model, the association between pornography use and depression/anxiety became non-significant, *b* = 0.03, *p* = .147 (see Table 5). In the case of adolescent women, a significant effect for pornography use was only observed in the Rijeka panel, *b* = 0.12, *p* < .001. This relationship remained significant even when controlling for the contribution of impulsiveness and family environment, *b* = 0.09, *p* = .007 (see Table 6).

**Table 5:** Linear Mixed modeling lagged models involving pornography use, depression and anxiety, and self-esteem in the males (*n* = 200 Zagreb; *n* = 468 Rijeka).

		*Later Depression and Anxiety*				*Later Self-Esteem*				*Later Pornography Use*			
		Zagreb Panel		Rijeka Panel		Zagreb Panel		Rijeka Panel		Zagreb Panel		Rijeka Panel	
		Basic Model (*b*)	Control Model (*b*)	Basic Model (*b*)	Control Model (*b*)	Basic Model (*b*)	Control Model (*b*)	Basic Model (*b*)	Control Model (*b*)	Basic Model (*b*)	Control Model (*b*)	Basic Model (*b*)	Control Model (*b*)
**Fixed Effects**													
	Intercept	1.58	0.09	3.57***	2.24***	6.06***	6.71***	3.67***	4.46***	1.64*	1.78*	1.15***	0.47
	Pornography Use	-0.02	-0.06	0.05*	0.04	0.01	0.02	-0.01	0.00	0.75***	0.75***	0.82***	0.80***
	Depression and Anxiety	0.77***	0.74***	0.64***	0.60***	-0.07	-0.08	-0.02	-0.01	0.00	0.01	0.00	-0.01
	Self-Esteem	0.01	0.03	-0.08***	-0.05*	0.66***	0.65***	0.79***	0.78***	-0.02	-0.02	-0.02	-0.00
	Impulsiveness	—	0.07*	—	0.03*	—	-0.05	—	-0.04**	—	0.01	—	0.03**
	Family Environment	—	0.16	—	0.16***	—	0.08	—	0.00	—	-0.09	—	0.01
	*s*^*2*^	*s*^*2*^	*s*^*2*^	*s*^*2*^	*s*^*2*^	*s*^*2*^	*s*^*2*^	*s*^*2*^	*s*^*2*^	*s*^*2*^	*s2*	*s*^*2*^
**Random Intercept**												
	*School / Class*	0.09	0.06	0.03	0.03	0.00^1^	0.00^1^	0.00	0.00	0.00^1^	0.00^1^	0.02	0.02
**Residuals**												
	*Time 2*	—	—	4.84***	4.85***	—	—	3.11***	3.05***	—	—	2.35***	2.31***
	*Time 3*	5.86***	5.74***	4.47***	4.17***	4.34***	4.39***	3.87***	3.84***	2.08***	2.04***	1.91***	1.92***
	*Time 4*	5.09***	4.86***	4.08***	4.08***	4.80***	4.74***	3.41***	3.39***	1.88***	1.89***	1.85***	1.82***
	*Time 5*	—	—	4.94***	4.73***	—	—	2.80***	2.77***	—	—	2.05***	2.03***
	*ARH1* ρ	-0.45***	-0.41***	-0.29***	-0.26***	-0.11	-0.09	-0.39***	-0.38***	-0.16	-0.17	-0.27***	-0.26***

^1^ In these models, the random intercept was fixed to zero to allow for model convergence.

* *p* < .05

** *p* < .01

*** *p* < .001

**Table 6:** Linear mixed modeling lagged models involving pornography use, depression and anxiety, and self-esteem in females (*n* = 443 Zagreb; *n* = 711 Rijeka).

		*Later Depression and Anxiety*				*Later Self-Esteem*				*Later Pornography Use*			
		Zagreb Panel		Rijeka Panel		Zagreb Panel		Rijeka Panel		Zagreb Panel		Rijeka Panel	
		Basic Model (*b*)	Control Model (*b*)	Basic Model (*b*)	Control Model (*b*)	Basic Model (*b*)	Control Model (*b*)	Basic Model (*b*)	Control Model (*b*)	Basic Model (*b*)	Control Model (*b*)	Basic Model (*b*)	Control Model (*b*)
**Fixed Effects**													
	Intercept	5.75***	4.39***	3.76***	2.44***	3.01***	4.06***	3.77***	5.03***	0.53	0.45	0.35*	0.05
	Pornography Use	0.06	0.04	0.12***	0.09**	0.05	0.06	0.04	0.05*	0.74***	0.73***	0.83***	0.82***
	Depression and Anxiety	0.59***	0.55***	0.64***	0.61***	-0.05*	-0.03	-0.05***	-0.04**	0.02	0.01	0.01	0.00
	Self-Esteem	-0.14***	10.11	-0.07***	-0.05**	0.82***	0.80***	0.79***	0.76***	-0.01	-0.00	-0.01	-0.00
	Impulsiveness	—	0.01	—	0.04**	—	-0.04*	—	-0.05***	—	-0.01	—	0.01
	Family Environment	—	0.24***	—	0.11**	—	-0.05	—	0.00	—	0.04	—	0.05***
		*s*^*2*^	*s*^*2*^	*s*^*2*^	*s*^*2*^	*s*^*2*^	*s*^*2*^	*s*^*2*^	*s*^*2*^	*s*^*2*^	*s*^*2*^	*s2*	*s*^*2*^
**Random Intercept**													
	*School / Class*	0.00^1^	0.00^1^	0.04	0.04	0.00	0.00^1^	0.03	0.03	0.02	0.02	0.01	0.01
**Residuals**													
	*Time 2*	—	—	5.42***	5.27***	—	—	3.92***	3.84***	—	—	1.11***	1.10***
	*Time 3*	5.94***	5.94***	5.58***	5.47***	4.33***	4.26***	3.80***	3.70***	1.63***	1.62***	0.97***	0.95***
	*Time 4*	6.90***	6.55***	6.18***	6.07***	5.49***	5.42***	3.62***	3.59***	1.44***	1.45***	1.26***	1.26***
	*Time 5*	—	—	5.65***	5.55***	—	—	3.25***	3.18***	—	—	0.93***	0.92***
	*ARH1* ρ	-0.20**	-10.18**	-0.28***	-0.26***	-0.49***	-0.40***	-0.35***	-0.34***	-0.19*	-0.19*	-0.36******	-0.35***

^1^ In these models, the random intercept was fixed to zero to allow for model convergence.

* *p* < .05

** *p* < .01

*** *p* < .001

The same approach was used to determine if pornography use at a given wave was associated with subsequent changes in adolescent self-esteem. No significant associations between pornography use and self-esteem were found among male or female adolescents in either the Zagreb (*b* = 0.01, *p* = .924 and *b* = 0.05, *p* = .153, respectively) or Rijeka panels (*b* = -0.01, *p* = .534 and *b* = 0.04, *p* = .139, respectively) (see Tables 5 and 6). Controlling for differences in impulsiveness and adverse family environment did not change this pattern of findings for males, but a positive association between pornography use and self-esteem, *b* = 0.05, *p* = .043, emerged among adolescent women in the Rijeka panel.

When the direction of prediction was reversed (from the indicators of psychological well-being to pornography use), neither symptoms of dysregulated mood nor self-esteem significantly predicted later pornography use in men and women from the two panel samples (all *p* > .05; see Tables 5 & 6). Adding impulsiveness and adverse family environment to the models did not affect these findings.

## Discussion

Reflecting concerns over adolescents’ use of online pornography, which include fears that immersion in pornographic imagery of impersonal sex and hypersexualized bodies may have negative consequences for young people’s psychological health, this study set out to assess temporal relationships between pornography use and various aspects of adolescent mental well-being. A combination of cross-lagged path analytic models and lagged linear mixed models were employed to examine these concerns in two independent samples of adolescent Croatian men and women. We did not find consistent evidence that pornography use was associated with negative changes in subjective well-being, symptoms of depression and anxiety, or self-esteem in either gender. Such findings are at odds with some interpretations of the available evidence from cross-sectional research [13].

To determine if the frequency of pornography use was temporally related to subjective well-being in the period from middle to late adolescence, two independent cross-legged path analytic models were tested with separate panels of adolescents. In both cases, the two measurements were spaced 12 months apart, which allowed for enough time to address longer-term, rather than short-term, sequelae of pornography use. After controlling for adverse family environment and impulsivity, our results offer no evidence of a direct association between the initial frequency pornography use and subsequent changes in subjective well-being. This null-effect finding was consistent across gender and panel sample.

In the second part of our analyses, which focused on two specific indicators of subjective well-being using lagged linear mixed modeling, we found no evidence of associations between pornography use and changes in subsequent self-esteem and dysregulated mood in adolescent men across the two panels. The same was true with respect to adolescent women in the Zagreb panel, however, among women in the larger Rijeka panel, pornography use was associated with increases in both self-esteem as well as symptoms of depression and anxiety. Focusing on patterns of consistent associations and null effects across samples, the current study does not support the notion that pornography use directly contributes to the dynamics of mental well-being in mid to late adolescence.

Although the current study failed to find consistent direct temporal effects between pornography use and subjective well-being across two independent samples, several inconsistent effects among female adolescents were observed. As noted above, lagged linear mixed modeling indicated that earlier pornography use was associated with increases in both self-esteem and symptoms of depression and anxiety among adolescent women in the Rijeka but not the Zagreb panel. One possibility, of course, is that this inconsistency occurred because the associations between pornography use and these indicators of well-being were too small to be detected in the Zagreb sample, which had fewer participants than the Rijeka sample. If this is the case, the presumed impact of pornography use on adolescent women’s depression and anxiety appears to be balanced by an association between pornography use and increases in self-esteem, which may help to explain why the effect of pornography use does not appear to carry over to global measures of subjective well-being among women. It is important to note that these seemingly opposing relationships may represent independent patterns of association that are present in different subsets of women in the Rijeka sample. That is, it is not necessarily the case that women who experienced increases in depression and anxiety also experienced increases in self-esteem.

At present, the mechanisms underlying these observed relationships remain unclear. Although these associations may represent causal impacts of pornography use on specific facets of female adolescents’ mental well-being, it is also possible that one or both of these effects may represent spurious correlations caused by unmeasured variables that covary with both pornography use and these indicators. One often overlooked example in pornography research is sex-drive [49,50]—which may have particular applicability to symptoms of depression and anxiety among women, as sexual interest is almost invariably stigmatized when exhibited by young women (i.e., “slut shaming”). Given the lack of consistency in these findings, additional research is recommended before strong conclusions are drawn about the associations between pornography use and mental well-being of adolescent women.

The findings also indicated a small and inconsistent (observed only in the Zagreb panel) contribution of adolescent women’s low initial subjective well-being on growth in pornography use over the next 12 months. This finding is similar to results reported by Peter and Valkenburg [11] who found that lower antecedent life satisfaction among adolescents was associated with subsequent increases in pornography viewing over time and is consistent with self-reported motivation for pornography use to alleviate negative affective states [32–35]. Unfortunately, Peter and Valkenburg did not consider whether the relationship in question was more prominent among women than men, so the current results are not directly comparable. Further, the failure to replicate the finding in the larger of our two panels suggests possible spuriousness. Without further confirmatory research, the finding should be considered tentative.

The pattern of significant covariances in the cross-lagged models focusing on subjective well-being is also noteworthy. The bi-directional associations between pornography use and subjective well-being at T1 were inconsistent across panels in both adolescent men and adolescent women. For this reason, we are skeptical of the suppression effect noted in males in the Rijeka panel, which indicated that pornography use was positively associated with subjective well-being once impulsiveness and family environment were controlled for. While certainly unexpected, and potentially interesting, it may not replicate in other studies. In contrast, a consistent bi-directional association between pornography use and subjective well-being at T2 was observed among adolescent men from both cities. Coupled with a systematic absence of directed paths between these constructs, this finding suggests the existence of other unassessed constructs that affect both pornography use (positively) and well-being (negatively). One example might be the timing of pubertal development, with an early onset—which is thought to explain some of the links between adolescent sexual behavior and emotional distress more generally [51]—being of particular importance.

It is important to locate the current findings within the specific sociocultural context in which the samples were drawn. It is likely that cultural attitudes towards sexuality—particularly towards adolescent sexuality—may shape pornography use and its consequences among adolescents. It is interesting to note that while the current data were drawn from a highly religious Eastern European country [52,53], the prevalence rates of pornography use (Zagreb, 50%; Rijeka 56%) were at the higher end of range of published prevalence estimates, (7–59%)[12]. Unfortunately, it’s difficult to know what to make of such figures. Cross-cultural comparisons of pornography use among adolescents are hindered by differences in operational definitions across studies, differences in sample age ranges, and by differences in the technical means of accessing pornography between samples—which varies not only by region but also by year in which the research was conducted. Nevertheless, it is curious that current study failed to find strong consistent negative associations between pornography use and wellbeing in a Roman Catholic country with a high-profile and publicly visible sexual conservative movement [54,55]. This is precisely the social context in which one would expect high guilt and anxiety surrounding pornography use [56,57], which is known to be related to poor mental wellbeing. The extent that the current findings generalize beyond the Croatian context remains unknown, and the conclusions reached in this paper should be considered tentative until replications occur in other cultures.

Finally, it should be noted that the average impulsiveness scores were consistently higher in female than male participants in both panel samples. At first glance, this may seem unexpected and indicative of problematic measurement. The evidence on sex differences in impulsivity is, however, mixed [58]. While impulsiveness has been found to be higher among boys than girls in childhood, the difference seems to wane in adolescence, most likely due to a combination of developmental, hormonal and normative influences [59]. Actually, a recent meta-analysis suggested that adolescent women may be more vulnerable to impulsivity-associated risk taking [60]. Routinely observed higher levels of risk taking among adolescent men may primarily reflect sensation seeking [61], which is systematically higher in men than women, rather than the related but independent construct of impulsivity [62]. What complicates conclusions regarding this study’s findings of inconsistent but similarly sized association between impulsivity and well-being in male and female adolescents, is the consensus about impulsiveness as a heterogeneous trait [60–62]. The fact that the measure used in this study was essentially one-dimensional [45], tapping into general impulsivity, likely obscured gender-specific nuances [58,59].

### Study limitations

Apart from its strengths, such as being the first study to explore a possible association between adolescent pornography use and changes in well-being in two independent panel samples, several study limitations need to be considered when interpreting our findings. Although there is literature suggesting that single-item indicators are recommended when the measured construct and its attribute are easily and uniformly understood [63,64], our indicator of pornography use precluded the assessment of the related measurement error. Two additional limitations pertain to our study design. First, the assessment based on only two measurement points represents the most basic longitudinal exploration and precludes an exploration of the dynamics of cross-domain relationships or the assessment of the duration of an association. Secondly, although the number of male participants in the Zagreb panel appears sufficiently large for multi-group structural equation modeling [46], the related statistical power to detect weak associations observed in the Zagreb panel was certainly restricted [65].

### Conclusions

Despite common public concerns that surround adolescent use of sexual media [66], the results of this first longitudinal assessment of the relationship between pornography use and adolescents’ subjective well-being provide no evidence that pornography use contributes to decreased subjective well-being in adolescent men. We found, however, limited evidence of the contradictory contribution of pornography use to female adolescents’ dysregulated mood and self-evaluation. Future research in this area should use large-scale prospective designs, which would include different developmental stages, to clarify possible effects in adolescent women. Given the public concern surrounding pornography use among adolescents, the veracity of these findings will likely be challenged. Thus, replication of our findings with diverse adolescent samples from other cultural settings is highly warranted.

## Supporting information

S1 Appendix Study measures. (DOCX)

## References

[1] Kendrick W. The secret museum: Pornography in modern culture New York: Viking; 1987.

[2] Cooper A, Delmonico DL, Burg R. Cybersex users, abusers, and compulsives: New findings and implications. Sex Addict Compulsivity. 2000;7: 5–29. 10.1080/10720160008400205

[3] Cooper A, Delmonico DL, Griffin-Shelley E, Mathy RM. Online Sexual Activity:An Examination of Potentially Problematic Behaviors. Sex Addict Compulsivity. 2004;11: 129–143. 10.1080/10720160490882642

[4] Byers LJ, Menzies KS, O’Grady WL. The impact of computer variables on the viewing and sending of sexually explicit material on the Internet: Testing Cooper’s “Triple-A Engine.” Can J Hum Sex. 2004;13: 157–169.

[5] Dines G. Is porn immoral? That doesn’t matter: It’s a public health crisis. The Washington Post. 4 2016.

[6] Emba C. Is pornography a public health crisis? In: Washington Post [Internet]. 2016 [cited 8 Feb 2018]. Available: https://www.washingtonpost.com/news/in-theory/wp/2016/05/23/porn-you-know-it-when-you-see-it-but-should-it-be-regulated/?utm_term=.b64d4a1f0f7b

[7] Standing Committee on Health. Report on the public health effects of the ease of access and viewing of online violent and degrading sexually explicit material on children, women and men. 2017.

[8] Petley J. The regulation of pornography on video-on-demand in the United Kingdom. Porn Stud. Taylor & Francis; 2014;1: 260–284. 10.1080/23268743.2014.927705

[9] Kohut T. An Empirical Investigation of the Conccept of Pornography Univesity of Western Ontario 2014.

[10] Short MB, Black L, Smith AH, Wetterneck CT, Wells DE. A Review of Internet Pornography Use Research: Methodology and Content from the Past 10 Years. Cyberpsychology, Behav Soc Netw. 2012;15: 13–23. 10.1089/cyber.2010.0477 22032795

[11] Peter J, Valkenburg PM. The use of sexually explicit internet material and its antecedents: A longitudinal comparison of adolescents and adults. Arch Sex Behav. 2011;40: 1015–1025. 10.1007/s10508-010-9644-x 20623250 PMC3180617

[12] Peter J, Valkenburg PM. Adolescents and Pornography: A Review of 20 Years of Research. J Sex Res. Routledge; 2016;53: 509–531. 10.1080/00224499.2016.1143441 27105446

[13] Collins RL, Strasburger VC, Brown JD, Donnerstein E, Lenhart A, Ward LM. Sexual Media and Childhood Well-being and Health. Pediatrics. 2017;140: S162–S166. 10.1542/peds.2016-1758X 29093054

[14] Pizzol D, Bertoldo A, Foresta C. Adolescents and web porn: A new era of sexuality. Int J Adolesc Med Health. 2016;28: 169–173. 10.1515/ijamh-2015-0003 26251980

[15] Owens EW, Behun RJ, Manning JC, Reid RC. The Impact of Internet Pornography on Adolescents: A Review of the Research. Sex Addict Compulsivity. 2012;19: 99–122. 10.1080/10720162.2012.660431

[16] La Placa V, McNaught A, Knight A. Discourse on wellbeing in research and practice. Int J Wellbeing. 2013;3: 116–125. 10.5502/ijw.v3i1.7

[17] Dodge R, Daly A, Huyton J, Sanders L. The challenge of defining wellbeing. Int J Wellbeing. 2012;2: 222–235. 10.5502/ijw.v2i3.4

[18] Deci EL, Ryan RM. Hedonia, eudaimonia, and well-being: An introduction. J Happiness Stud. 2008;9: 1–11. 10.1007/s10902-006-9018-1

[19] Diener E, Suh EM, Lucas RE, Smith H. Subjective well-being: three decades of progress. Psychol Bull. 1999;125: 276–302.

[20] Ryff CD. Happiness is everything, or is it? Explorations on the meaning of psychological well-being. J Pers Soc Psychol. 1989;57: 1069–1081. 10.1037/0022-3514.57.6.1069

[21] Doornwaard SM, Bickham DS, Rich M, Vanwesenbeeck I, van den Eijnden RJJM, ter Bogt TFM. Sex-Related Online Behaviors and Adolescents’ Body and Sexual Self-Perceptions. Pediatrics. 2014;134: 1103–1110. 10.1542/peds.2014-0592 25404728

[22] Morrison TG, Ellis SR, Morrison MA, Bearden A, Harriman RL. Exposure to sexually explicit material and variations in body esteem, genital attitudes, and sexual esteem among a sample of Canadian men. J Mens Stud. 2006;14: 209–222.

[23] Philaterou AG, Mahfouz A, Allen KR. Use of Internet pornography and men’s well-being. Int J Mens Health. 2005;4: 149–169.

[24] Tylka TL. No harm in looking, right? Men’s pornography consumption, body image, and well-being. Psychol Men Masculinity. 2015;16: 97–107. 10.1037/a0035774

[25] Weaver JB, Weaver SS, Mays D, Hopkins GL, Kannenberg W, MCBride D. Mental- and physical-health indicators and sexually explicit media use behavior by adults. J Sex Med. 2011;8: 764–772. 10.1111/j.1743-6109.2010.02030.x 20946159

[26] Kim Y-H. Adolescents ‘ Health Behaviours and Its Associations With Psychological Variables. Cent Eur J Public Health. 2011;19: 205–209. 22432395 10.21101/cejph.a3694

[27] Ybarra ML, Mitchell KJ, Hamburger M, Diener-West M, Leaf PJ. X-rated material and perpetration of sexually aggressive behavior among children and adolescents: Is there a link? Aggress Behav. 2011;37: 1–18. 10.1002/ab.20367 21046607

[28] Kim Y-H. Korean adolescents’ health risk behaviors and their relationships with the selected psychological constructs. J Adolesc Heal. 2001;29: 298–306. 10.1016/s1054-139x(01)00218-x 11587914

[29] Morrison TG, Bearden A, Harriman RL, Morrison MA, Ellis SR. Correlates of exposure to sexually explicit material among Canadian post-secondary students. Can J Hum Sex. 2004;13: 143–156. Available: http://www.scopus.com/inward/record.url?eid=2-s2.0-17644365811&partnerID=tZOtx3y1

[30] Popovic M. Pornography Use and Closeness with Others in Men. Arch Sex Behav. 2011;40: 449–456. 10.1007/s10508-010-9648-6 20652735

[31] Wetterneck CT, Burgess AJ, Short MB, Smith AH, Cervantes ME. The role of sexual compulsivity, impulsivity, and experiential avoidance in internet pornography use. Psychol Rec. 2012;62: 3–18. 10.1007/BF03395783

[32] Bridges AJ, Morokoff PJ. Sexual media use and relational satisfaction in heterosexual couples. Pers Relatsh. 2011;18: 562–585. 10.1111/j.1475-6811.2010.01328.x

[33] Hempel K. Dirty girls come clean: The experience of women who use sexually explicit materials and its link to their spirituality. Institute of Transpersonal Psychology. 2012.

[34] Lawrence KA, Herold ES. Women’s attitudes toward and experience with sexually explicit materials. J Sex Res. 1988;24: 161–169. 10.1080/00224498809551406 22375643

[35] Perse EM. Uses of Erotica and Acceptance of Rape Myths. Communic Res. 1994;21: 488–515. 10.1177/009365094021004003

[36] Valkenburg PM, Peter J. The differential susceptibility to media effects model. J Commun. 2013;63: 221–243. 10.1111/jcom.12024

[37] Keyes CLM, Shmotkin D, Ryff CD. Optimizing well-being: The empirical encounter of two traditions. J Pers Soc Psychol. 2002;82: 1007–1022. 10.1037//0022-3514.82.6.1007 12051575

[38] Schimmack U, Diener E. Predictive validity of explicit and implicit self-esteem for subjective well-being. J Res Pers. 2003;37: 100–106. 10.1016/S0092-6566(02)00532-9

[39] Chen H, Cohen P, Chen S. How big is a big odds ratio? Interpreting the magnitudes of odds ratios in epidemiological studies. Commun Stat Simul Comput. 2010;39: 860–864. 10.1080/03610911003650383

[40] Kolesarić V, Ajduković M, editors. Etički kodeks istraživanja s djecom Zagreb: Vijeće za djecu Vlade Republike Hrvatske; 2003.

[41] Proctor P. Subjective well-being In: Michalos AC, editor. Encyclopedia of quality of life and well-being research. Dordrecht: Springer; 2014 pp. 6437–6441.

[42] Tomyn AJ, Cummins RA. The Subjective Wellbeing of High-School Students: Validating the Personal Wellbeing Index-School Children. Soc Indic Res. 2011;101: 405–418. 10.1007/s11205-010-9668-6

[43] Kroenke K, Spitzer RL, Williams JBW, Lowe B. An Ultra-Brief Screening Scale for Anxiety and Depression: The PHQ-4. Psychosomatics. 2009;50: 613–621. 10.1176/appi.psy.50.6.613 19996233

[44] Cénat JM, Hébert M, Blais M, Lavoie F, Guerrier M, Derivois D. Cyberbullying, psychological distress and self-esteem among youth in Quebec schools. J Affect Disord. 2014;169: 7–9. 10.1016/j.jad.2014.07.019 25128859 PMC4679325

[45] Steinberg L, Sharp C, Stanford MS, Tharp AT. New tricks for an old measure: The development of the Barratt Impulsiveness Scale-Brief (BIS-Brief). Psychol Assess. 2013;25: 216–226. 10.1037/a0030550 23148649

[46] Little TD. Longitudinal structural equation modeling New York: Guilford; 2013.

[47] Meade AW, Johnson EC, Braddy PW. Power and sensitivity of alternative fit indices in tests of measurement invariance. J Appl Psychol. 2008;93: 568–592. 10.1037/0021-9010.93.3.568 18457487

[48] Graham JW. Missing Data: Analysis and Design New York: Springer; 2012.

[49] Baer JL, Kohut T, Fisher WA. Is pornography use associated with anti-woman sexual aggression? Re-examining the Confluence Model with third variable considerations. Can J Hum Sex. 2015;24: 160–173. 10.3138/cjhs.242-A6

[50] Campbell L, Kohut T. The use and effects of pornography in romantic relationships. Curr Opin Psychol. Elsevier Ltd; 2017;13: 6–10. 10.1016/j.copsyc.2016.03.004 28813295

[51] Harden KP. A Sex-Positive Framework for Research on Adolescent Sexuality. Perspect Psychol Sci. 2014;9: 455–469. 10.1177/1745691614535934 26186753

[52] Štulhofer A, Rimac I. Determinants of homonegativity in Europe. J Sex Res. Taylor & Francis Group; 2009;46: 24–32. 10.1080/00224490802398373 19012059

[53] Luijkx R, Halman L, Sieben I, Brislinger E, Quandt M. European Values in Numbers Trends and Traditions at the Turn of the Century. 2016 10.1163/9789004328525

[54] Zagreb AP in. Croatians vote to ban gay marriage. The Guardian. 12 2013.

[55] Staff R. Croatians protest against European treaty they say threatens traditional family. Reuters. Mar 2018.

[56] Grubbs JB, Perry SL. Moral Incongruence and Pornography Use: A Critical Review and Integration. 2017; 10.1080/00224499.2018.1427204 29412013

[57] Kohut T, Štulhofer A. The Role of Religiosity in Adolescents’ Compulsive Pornography Use: A Longitudinal Assessment. J Sex Marital Ther. 2018; 10.1080/0092623X.2018.1466012 29676698

[58] Mitchell MR, Potenza MN. Importance of sex differences in impulse control and addictions. Front Psychiatry. 2015;6: 27–30. 10.3389/fpsyt.2015.00027 25762943 PMC4332159

[59] Weinstein A, Dannon P. Is Impulsivity a Male Trait Rather than Female Trait? Exploring the Sex Difference in Impulsivity. Curr Behav Neurosci Reports. 2015;2: 9–14. 10.1007/s40473-015-0031-8

[60] Dir AL, Coskunpinar A, Cyders MA. A meta-analytic review of the relationship between adolescent risky sexual behavior and impulsivity across gender, age, and race. Clin Psychol Rev. Elsevier Ltd; 2014;34: 551–562. 10.1016/j.cpr.2014.08.004 25261740

[61] Romer D. Adolescent Risk Taking, Impulsivity, and Brain Development: Implications for Prevention. Dev Psychobiol. 2012;52: 263–276. 10.1002/dev.20442.Adolescent PMC3445337 20175097

[62] Cross CP, Copping LT, Campbell A. Sex Differences in Impulsivity: A Meta-Analysis. Psychol Bull. 2011;137: 97–130. 10.1037/a0021591 21219058

[63] Bergkvist L, Rossiter JR. The Predictive Validity of Multiple-Item Versus Single-Item Measures of the Same Constructs. J Mark Res. 2007;44: 175–184. 10.1509/jmkr.44.2.175

[64] Diamantopoulos A, Sarstedt M, Fuchs C, Wilczynski P, Kaiser S. Guidelines for choosing between multi-item and single-item scales for construct measurement: A predictive validity perspective. J Acad Mark Sci. 2012;40: 434–449. 10.1007/s11747-011-0300-3

[65] Wolf EJ, Harrington KM, Clark SL, Miller MW. Sample Size Requirements for Structural Equation Models: An Evaluation of Power, Bias, and Solution Propriety. Natl Institutes Heal. 2013;76: 913–934. 10.1177/0013164413495237 Sample 25705052 PMC4334479

[66] Levine J. Harmful to minors: The perils of protecting children from sex University of Minnesota Press; 2006.

