# Supplementary material for: Is pornography use a risk for adolescent well-being? An examination of temporal relationships in two independent panel samples
Source: PLoS One. 2018 Aug 10;13(8):e0202048. doi: 10.1371/journal.pone.0202048 (PMC6088458; doi:10.1371/journal.pone.0202048)
Supplement: S1 Appendix — (DOCX) [file pone.0202048.s001.docx]

**S1 Appendix. Study Measures**

**Measures in Croatian:**

**Pornography use:**

U posljednjih 6 mjeseci, koliko si često koristio/la pornografiju? ⇨ *SAMO JEDAN ODGOVOR*

1 – Niti jednom

2 – Nekoliko puta

3 – Otprilike jednom mjesečno

4 – 2-3 puta mjesečno

5 – Otprilike jednom tjedno

6 – Nekoliko puta tjedno

7 – Svakodnevno ili gotovo svakodnevno

8 – Više puta dnevno

**Subjective well-being**

|  | Željeli bismo te pitati koliko si zadovoljan/a sa: | 1 = IZRAZITO NEZADOVOLJAN  ….  10 = POTPUNO ZADOVOLJAN | | | | | | | | | |
| --- | --- | --- | --- | --- | --- | --- | --- | --- | --- | --- | --- |
| **1** | Svojim životom u cjelini. | 1 | 2 | 3 | 4 | 5 | 6 | 7 | 8 | 9 | 10 |
| **2** | Svojim zdravljem. | 1 | 2 | 3 | 4 | 5 | 6 | 7 | 8 | 9 | 10 |
| **3** | Onim što si do sada postigao/la. | 1 | 2 | 3 | 4 | 5 | 6 | 7 | 8 | 9 | 10 |
| **4** | Svojim odnosima s ljudima. | 1 | 2 | 3 | 4 | 5 | 6 | 7 | 8 | 9 | 10 |

**Depression and anxiety**

| TIJEKOM PROTEKLA DVA TJEDNA, koliko ti se često dogodilo nešto od sljedećeg? | UOPĆNE NE | NEKOLIKO DANA | VIŠE OD POLOVINE VREMENA (VIŠE OD 7 DANA) | GOTOVO SVAKI DAN |
| --- | --- | --- | --- | --- |
| Osjećao/la sam se nervozno ili napeto. | 1 | 2 | 3 | 4 |
| Nisam se mogao/la prestati brinuti ili mogao/la kontrolirati zabrinutost. | 1 | 2 | 3 | 4 |
| Nisu me baš zanimale ni veselile stvari koje sam radio/la. | 1 | 2 | 3 | 4 |
| Osjećao/la sam se potišteno, loše raspoloženo ili beznadno. | 1 | 2 | 3 | 4 |

**Self-esteem**

| Procijeni koliko se sljedeće tvrdnje odnose na tebe: | UOPĆE SE **NE** ODNOSI NA MENE | UGLAVNOM SE **NE** ODNOSI NA MEME | NITI SE ODNOSI NITI SE NE ODNOSI | UGLAVNOM SE ODNOSI NA MENE | U POTPUNOSTI SE ODNOSI NA MENE |
| --- | --- | --- | --- | --- | --- |
| Općenito volim sebe takvog kakav jesam / takvu kakva jesam. | 1 | 2 | 3 | 4 | 5 |
| U cjelini, mogu se ponositi sobom. | 1 | 2 | 3 | 4 | 5 |
| Imam mnogo vrlina | 1 | 2 | 3 | 4 | 5 |
| Kada nešto radim, dobro to radim. | 1 | 2 | 3 | 4 | 5 |

**Impulsiveness**

| U kojoj se mjeri sljedeće tvrdnje odnose na tebe? | NIKADA / RIJETKO | POVREMENO | ČESTO | GOTOVO UVIJEK / UVIJEK |
| --- | --- | --- | --- | --- |
| Radim stvari bez razmišljanja. | 1 | 2 | 3 | 4 |
| Ne planiram stvari unaprijed. | 1 | 2 | 3 | 4 |
| Dobro vladam sobom. | 1 | 2 | 3 | 4 |
| Kažem stvari bez razmišljanja. | 1 | 2 | 3 | 4 |
| Smireno razmišljam. | 1 | 2 | 3 | 4 |
| Reagiram naglo, bez razmišljanja. | 1 | 2 | 3 | 4 |

**Family environment**

|  | U posljednjih 12 mjeseci, koliko često je u tvojoj obitelji bilo: | NIJEDNOM | RIJETKO | PONEKAD | ČESTO |
| --- | --- | --- | --- | --- | --- |
| **1** | Žestokih svađa | 1 | 2 | 3 | 4 |
| **2** | Ponašanja kao što su odgurivanje, razbijanje predmeta, prijetnje... | 1 | 2 | 3 | 4 |
| **3** | Ignoriranja (dugotrajno odbijanje razgovora, ponašanje kao da drugi ne postoji) | 1 | 2 | 3 | 4 |

**English translation of measures:**

**Pornography use**

**How often have you used pornography during the last 6 months?**

1 – Not once

2 – Several times

3 – Once a month

4 – 2-3 times a month

5 – Once a week

6 – Several times a week

7 – Every day or almost every day

8 – Several times a day

**Subjective well-being**

| How satisfied are you… | | | | | | | | | |
| --- | --- | --- | --- | --- | --- | --- | --- | --- | --- |
| 1 | | …with your life in total? | | | | | | | |
| 2 | | …with your health? | | | | | | | |
| 3 | | …with what you have achieved so far? | | | | | | | |
| 4 | | …with your relationship with others? | | | | | | | |
| Extremely unsatisfied | |  |  |  |  |  |  | Completely satisfied | |
| 1 | 2 | 3 | 4 | 5 | 6 | 7 | 8 | 9 | 10 |

**Depression and anxiety**

|  | **During last two weeks, how often have you experienced...** | NOT AT ALL | SEVERAL DAYS | MORE THAN HALF OF THE DAYS | NEARLY EVERY DAY |
| --- | --- | --- | --- | --- | --- |
| **1** | Feeling nervous, anxious or on edge. | 1 | 2 | 3 | 4 |
| **2** | Not being able to stop or control worrying. | 1 | 2 | 3 | 4 |
| **3** | Little interest or pleasure in doing things. | 1 | 2 | 3 | 4 |
| **4** | Feeling down, depressed, or hopeless. | 1 | 2 | 3 | 4 |

**Self-esteem**

|  | **Estimate do the following statements relate to you:** | IT DOES NOT RELATE TO ME AT ALL | IT DOES NOT RELATE TO ME | NOR IT RELATES TO ME NOR IT DOESNT | IT RELATES TO ME | IT RELATES TO ME COMPLETELY |
| --- | --- | --- | --- | --- | --- | --- |
| **1** | In general, I like myself the way I am | 1 | 2 | 3 | 4 | 5 |
| **2** | Overall, I have a lot to be proud of | 1 | 2 | 3 | 4 | 5 |
| **3** | I have a lot of qualities | 1 | 2 | 3 | 4 | 5 |
| **4** | When I do something, I do it well | 1 | 2 | 3 | 4 | 5 |

**Impulsiveness**

|  | **Estimate how often do the following statements relate to you:** | NEVER / RARELY | OCCASIONALLY | OFTEN | ALMOST ALWAYS / ALWAYS |
| --- | --- | --- | --- | --- | --- |
| **1** | I don’t plan tasks carefully. | 1 | 2 | 3 | 4 |
| **2** | I do things without thinking. | 1 | 2 | 3 | 4 |
| **3** | I am self-controlled. | 1 | 2 | 3 | 4 |
| **4** | I am a careful thinker. | 1 | 2 | 3 | 4 |
| **5** | I say things without thinking. | 1 | 2 | 3 | 4 |
| **6** | I act on the spur of the moment. | 1 | 2 | 3 | 4 |

**Family environment**

|  | **In the last 12 months, how often in your family were there:** | NOT ONCE | RARELY | SOMETIMES | OFTEN |
| --- | --- | --- | --- | --- | --- |
| **1** | Intense quarrels | 1 | 2 | 3 | 4 |
| **2** | Violent behavior (showing, breaking stuff, slapping…) | 1 | 2 | 3 | 4 |
| **3** | Ignoring (refusing a conversation, acting like the other doesn’t exist) | 1 | 2 | 3 | 4 |
